# Reducing number entry errors: solving a widespread, serious problem

**DOI:** 10.1098/rsif.2010.0112

**Published:** 2010-04-07

**Authors:** Harold Thimbleby, Paul Cairns

**Affiliations:** 1 Future Interaction Technology Laboratory, Swansea University, Swansea SA2 8PP, UK; 2 Department of Computer Science, University of York, York YO10 5DD, UK

**Keywords:** number entry, human error, dependable systems, user interfaces

## Abstract

Number entry is ubiquitous: it is required in many fields including science, healthcare, education, government, mathematics and finance. People entering numbers are to be expected to make errors, but shockingly few systems make any effort to detect, block or otherwise manage errors. Worse, errors may be ignored but processed in arbitrary ways, with unintended results. A standard class of error (defined in the paper) is an ‘out by 10 error’, which is easily made by miskeying a decimal point or a zero. In safety-critical domains, such as drug delivery, out by 10 errors generally have adverse consequences. Here, we expose the extent of the problem of numeric errors in a very wide range of systems. An analysis of better error management is presented: under reasonable assumptions, we show that the probability of out by 10 errors can be halved by better user interface design. We provide a demonstration user interface to show that the approach is practical.

To kill an error is as good a service as, and sometimes even better than, the establishing of a new truth or fact.

(Charles Darwin 1879 [2008], p. 229)

## 1. Introduction

At first sight, typing numbers is such a mundane task that it seems not to merit a second glance. Naturally, when it comes to entering numbers, humans are prone to make errors, but—astonishingly—many systems make no effort to detect or manage possible errors, causing incorrect and unpredictable results. This paper exposes the extent of this problem in a wide range of systems. We show that the problem cannot be dismissed merely by blaming the user: indeed, we show that some system logs, which might otherwise be thought of as a formal record of user actions, cannot be relied on to assign blame.

Systems should be designed to manage errors, as errors will always eventually occur regardless of user skill or training. We therefore show how better designs for number entry may be approached; we present a new, improved user interface for preventing many number entry errors, and we argue that the new approach can approximately halve the probability of an important class of adverse events arising from number entry error.

We note that problems with complex software are widely recognized (Leveson 1995; Fox *et al.* 2009; Hoare 2009; Jackson 2009), but, to our knowledge, this article is the first to report the extent of serious problems with the seemingly trivial issue of processing number entry.

## 2. Widespread problems with real systems

**Figure 1.**
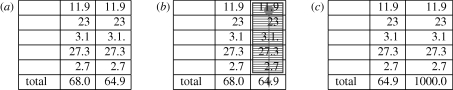
Errors in adding numbers in Microsoft Excel. Excel's SUM() function, which is used to total all columns in this figure, ignores values that are not numbers. No errors are reported in any of the examples. (*a*) Two apparently identical sums giving different results. The erroneous sum in the right-hand column is caused by 3.1. having a final decimal point/full stop, and hence being treated as text, and thus processed as zero by SUM. The difference between the column sums may not be noticed by a user, particularly since in normal use they are unlikely to double-check the ‘same’ columns, as used here for illustrative purposes. (*b*) The ‘show precedents’ feature is one way to help check calculations. It highlights the operands of a cell, but here the precedents for the incorrect total are shown as *including* the value that has been ignored. Evidently, Excel's notion of ‘precedents’ is the range of *possible* operands, rather than the *actual* operands, and therefore the feature is misleading. (*c*) Through innocent error or intentional mischief, even more unusual column sums can be produced. In the left column, the cell ‘3.1’ is generated by the formula ='3.1', which turns the apparently correct number 3.1 into a string, with value zero as before. In the right column, the cell ‘23’ is actually the number 995, but formatted as ‘23’ using a custom format.

Entering numbers seems like an apparently routine task, but it is in fact less dependable than it appears. Figure 1*a* shows an everyday example, here taken from Microsoft Excel (or Apple Numbers; the two applications behave in essentially the same way for the purposes of this paper). Two columns of numbers are supposed to be added up. In figure 1, the column totals should be the same, but small typing errors make the totals incorrect without any warning, even though no user is likely to want things that look like numbers (e.g. ‘3.1’) to be treated as anything but the numbers they seem to be. Using Excel's ‘show precedents’ feature, there is no indication that there is a problem (see figure 1*b*). And with frankly devious use of the formatting functions, even greater errors are possible, as in figure 1*c*—though we note that it is very easy to lose track of formatting, and the type of error illustrated here could arise by accident and be very hard to track down.

The examples in figure 1 illustrate the problems: the errors, whether caused intentionally or through accidental slips, are not immediately obvious to a casual glance, though for illustrative purposes the examples are not so large that the totals cannot readily enough be done in one's head. Of course, in typical applications of Excel, the spreadsheets will be much larger, and errors will be correspondingly harder to find.

### 2.1. Human error

Human error can be broadly classified as violations, mistakes and slips (Reason 2008). If users intend to make an error in their spreadsheet, perhaps to claim higher expenses, this is a deliberate violation. This type of error is unlikely to be detected by a computer except probabilistically using pattern matching, which might reveal it as anomalous behaviour. Secondly, the user may have picked up the wrong receipts: they intend to enter correct numbers, but the ones entered are in fact erroneous; this is a mistake. Finally, the user may know and intend to enter the correct number, but they fumble or accidentally hit the wrong keys; this is a slip.

Although slips may produce errors that are syntactic errors, syntactic errors cannot necessarily be attributed to slips. For example, the error in figure 1*a* could arise for all sorts of reasons: perhaps the user thought ending numbers with full stops was innocuous, which would be a mistake; or the user wanted to conceal incorrect financial accounts, which would be a violation; or the user accidentally hit the decimal point again, which would be a slip. In any case, the consequence is that a syntactically incorrect number is processed as if it were a valid number.

Ironically, the more skilled a user, the less attention they will pay to what ought to be routine outcomes, so the more likely these types of error will go unnoticed until they have untoward consequences. The reason is, as users become skilled, they automate actions, so their attention can be used more selectively; thus as they become more skilled, they pay less attention to the display, whose routine behaviour they have learnt to expect (Wickens & Hollands 2000). Worse, many users will be very practised in handling typing errors in word processors, but the error-correction features (such as delete keys) of word processors typically do not work in the same way on other devices (we give examples below), so this will encourage transfer errors, which again ironically will be worse for more skilled IT users.

Fortunately, blocking the consequences of some errors can be done without interpreting the user's slips, goals or aims, as this paper will show.

### 2.2. Safety-critical applications of number entry

Although the problems are ubiquitous, to our knowledge affecting all areas using numbers, the hazards are perhaps most powerfully illustrated in drug delivery, an area where such errors may have clear and rapid adverse outcomes: injury or death may be the consequence of number entry errors.

Consider the following scenario:

The Alaris Infusion Pump involved was accidentally programmed for 68 mL/hr instead of the ordered rate of 6 • 8 mL/hr. […] The patient expired the next day. It is believed that, although the pump did not malfunction, inherent design flaws in the infusion pump may have contributed to this event.

(Food and Drug Administration 2009)

Investigation revealed that the pump keypad was designed in such a way that it required more force to press a decimal point than a digit and that it was easy to think that you had pressed it when in fact the machine had not registered it. Nor was there feedback. Thus, while the cause of the death was attributed to the nurse making an error, the machine did little to support the nurse, particularly given that human error must be acknowledged as a known aspect of using any machine.

The Graseby 3400, a typical and representative drug delivery system widely used in hospitals, has a variety of problems in number entry, including the following.

— If the user enters an erroneous number, it will be accepted without complaint, but it will be misinterpreted. Thus keying 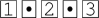 will enter 1 • 3.
— The Graseby 3400 user manual (Graseby Medical Ltd 2002) states that number entry works ‘like a calculator’, yet on calculators 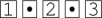 will generally enter 1 • 23.
— Entering 10 000 mg/h (i.e. keying 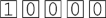 in the mg/h mode) is recorded by the Graseby as 100 • 00 mg/h.
— If the user takes more than approximately 4 s to enter a number, number entry silently resets. Thus, entering 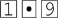 could enter either 1 • 90 or 9 • 00 in the mg/h mode. Users are unlikely to be familiar with this behaviour because it occurs rarely.
— If the dose units are changed (say from µg/h to ml/h), then the number being entered does not reset to zero and the user's keystrokes continue entering the number: so entering 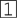 after a unit change could enter, say, 991 • 0 ml/h if 99 had been entered in the previous (µg/h) mode.
— Like many devices, the Graseby does not follow recommended practice for unit names (e.g. both the Institute for Safe Medication Practices (2006) and the National Patient Safety Agency (2010) recommend not using a lower case l for litre as it is confusable with the digit 1).

In *none* of the errors does the Graseby report any warning. Worse, if a user is in a busy situation where one of these problems occurs, they are surely also unlikely to be viewing the Graseby's LCD screen closely enough to confirm that the device is behaving as they intended. The Graseby is a safety-critical device that, we believe, ought to manage errors predictably and sensibly through blocking and giving the user an opportunity to correct errors. Like many devices it does neither. As table 1 indicates, many devices ignore errors and are inconsistent in the way they respond; table 2 shows how handling numbers *within* a single, typical device can be inconsistent. Some even change their behaviour depending on the number as it is entered: for example, on the Baxter Colleague, the decimal point disappears with values over 100, so 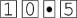 will enter 10 • 5, but 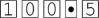 will enter 1005.

**Table 1.** Illustrative results from keying 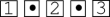 on various systems and devices. Note how different rules can produce the same result (e.g. iPhone Drug Infusion and Graseby 500) that for other input (e.g. 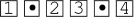) would give different results.

system	example	value	rationale
creatinine clearance calculator	Xeloda 71CRCL	123	despite the decimal point on the keypad, *all* data entry silently ignores it
search engine	Wolfram Alpha	6	treated as 1 × 2 × 3
office software	Microsoft Word tools calculate	1 • 5	treated as 1 • 2 + 0 • 3
infusion pump	Graseby 3400	1 • 3	dot zeros the decimal part, so 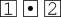 enters 1 • 0, then the 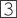 updates it to 1 • 3 (or 1 • 30 and 1 • 300 depending on mode)
handheld calculator	Casio HS-8V (Thimbleby 2005)	1 • 23	second and subsequent decimal points are ignored
mobile phone	iPhone DrugInfusion	1 • 2	second decimal point terminates number
infusion pump	Graseby 500	1 • 2	discards everything after first decimal digit
maths package	Wolfram Mathematica	0 • 36	treated as 1 • 2 × • 3
spreadsheet	Microsoft Excel	0	converted to a string, which may be treated as zero
spreadsheet	Sun OpenOffice	01/02/03	converted to a date—ambiguously, 1 February, 2 January, 3 February, 1901, 2001 … , etc.

It is clear that there is no systematic approach to user error, even for such a well-understood application as number entry. The resulting inconsistency between and, alarmingly, within systems (e.g. figure 1) will cause negative transfer (Wickens & Hollands 2000), which increases error rates. However, in some domains, there is clear guidance on number syntax; for example, the guidelines produced by the Institute for Safe Medication Practices (ISMP) (2006), adopted by the Food and Drug Administration (FDA) in 2006, provide specific guidelines for healthcare. Some relevant parts of the ISMP rules are summarized in table 3. The UK guidelines are similar (National Patient Safety Agency 2010).

Unfortunately, there are no clear data on how often keying errors are made in practice because, although many medical devices allow logging so that cases can be investigated, they log what the device interpreted, not what the person using the device keyed in. Thus, even in the earlier FDA scenario, it is conceivable that the nurse did in fact enter 6 • 8 by pressing 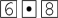, but, because of some unlogged internal state of the device, the number was taken to be 68. Another possibility is that the nurse perhaps keyed 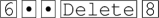 and the deliberate single delete, unnoticed by the user, deleted *both* decimal points—this would be similar behaviour to the iPhone pCalc calculator (Thomson 2009), which in this case would delete the decimal points *and* the preceding digit! Logs that record the result but not the user's exact actions (including timings for devices that have timeouts) are clearly inadequate to determine whether these or any other error or design confusion is the case; *conventional device logs are inadequate for investigations if the user is at risk of blame*.

## 3. A new user interface to prevent errors

**Figure 2.**
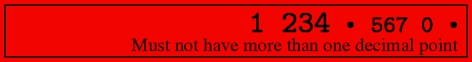
Snapshot of the error-blocking user interface after an error has occurred. The snapshot of the demonstration user interface shows handling a slip where the user has just entered a number with two decimal points (in our design, the device beeps and the screen also goes red to make the error more salient). An interactive demonstration is available.

We propose blocking the entry of numbers that do not conform to the ISMP guidance for the reliable formatting of numbers (table 3). We have built a demonstration user interface for number entry that shows how this works in practice and how the ideas could be implemented on many current devices (figure 2); a very similar user interface has also been made available in a drug dose calculator (Thimbleby 2008), which can be used on the Web or on the Apple iPhone as an example of a realistic device using the approach. The approach can be generalized to block numbers that fail any specified criteria. In fact, although not specified by ISMP, by way of illustration our demonstration blocks entry of numbers that are too long using a limit that can be changed. Such blocking is done very rarely by calculators, with resultant problems we avoid (see below and table 4).

The definition of the number entry interface is straightforward. The display shows exactly the character string that is in a buffer, which is initially empty. On each keystroke, the corresponding character is appended to the buffer; if the keystroke is 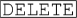 or 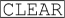, the buffer either has the last character, if any, deleted, or the entire buffer is cleared. After the buffer has been updated, it is parsed; there are then three cases:

1. The buffer is not a prefix of any valid number (e.g. the user has keyed two decimal points)—then a beep is made and an appropriate error message is displayed.
2. The buffer is not a valid number, but it is a prefix of a valid number (e.g. the user has entered ‘0 • ’, which must have more digits to follow the decimal point)—then a ‘continue’ symbol is shown.
3. The buffer is a valid number (e.g. the user has entered ‘2 • 3’ and they may key more)—an indicator is shown, perhaps by enabling or highlighting the 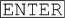 button.

There is an 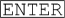 key, which on our demonstration represents the user ending the number; it is of course an error if 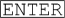 is pressed in cases (1) or (2) above. Finally, if the user does not correct an error but keys a digit or a decimal point, the buffer is extended and the error message is changed to specific instructions to delete or clear the error rather than a description of the number error itself.

Note that, in our approach, the buffer is initially empty, and the display shows nothing. In contrast, most number entry user interfaces initially display ‘0 • ’ or ‘0 • 0’ as the case may be, even when the user has entered nothing (table 2). Since the display does not change when either 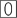 or 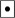 is keyed, this design creates a serious ambiguity: when the display shows ‘0 • ’ subsequently pressing 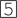 could display either ‘0 • 5’ or ‘5 • ’—which differ by a factor of 10. Our user interface uses a large decimal point and smaller digits following the decimal point, both recommended techniques to reduce the chance of misreading the display, as can be seen in figure 2.

**Table 2.** The Abbott Aimplus infusion pump illustrates two common problems of number entry with its varied response to the single key sequence 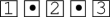. Pressing 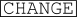 on the Abbot (or pressing 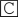 or equivalent on other devices) *immediately* before entering a number is crucial to the outcome. (*a*) When 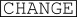 has just been pressed, the display is cleared, and 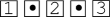 can only mean one thing, although exactly what depends on which mode the pump is in. (*b*) If 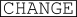 has not been pressed, the behaviour is almost arbitrary, and many values are possible depending on the user's prior keystrokes. Although the behaviour described in this table is specific to the Abbott Aimplus, like very many devices a display of ‘0 • ’ or ‘0 • 0’ is ambiguous, as it may be the result of pressing 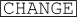, which resets the display, or it may be the result of previous keystrokes (such as 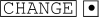, say, which produce *exactly* the same display but has a different effect on subsequent number entry).

(*a*) mode 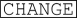 pressed	value	rationale
*µ*gm mL^−1^	123	decimal points are ignored
mL hr^−1^	1 • 2	ignores anything after one decimal digit
time	1.23 AM or 1.23 PM	in *some* time modes, decimal points change AM/PM. 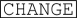 resets the time to 0.00 *but does not reset* AM/PM
(*b*) mode 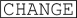 not pressed	possible value	rationale
mgm mL^−1^	9999 • 9	anything ignored unless 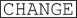 pressed first
mL hr^−1^	0 • 1	a user previously pressed, e.g. 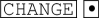
mL hr^−1^	0 • 0	a user previously pressed, e.g. 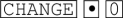
time	0.00 AM or 0.00 PM	it looks like 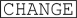 was pressed, but pressing 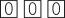 , etc., locks the display except for AM/PM, which still changes with the decimal point

It is important to note that our user interface makes no difference to users who make no keying errors; it simply blocks invalid numbers, which would typically be incorrectly handled by number entry systems.

Almost all interactive devices and certainly all PC applications have ample power and screen size to run our user interface; for many devices, nothing more would be required than an appropriate firmware update at their next service. If it is too hard to update the firmware, this in itself must be considered a design problem, as it has been well known at least since the 1980s that software needs revision, most particularly for safety-critical devices where unfixed bugs could have dangerous consequences. Single-line seven-segment digit displays would also be problematic, but these make decimal points inconspicuous and are therefore problematic for other reasons too.

## 4. Quantifying the benefits of preventing errors

To analyse the potential impact of this form of number entry interface, we consider the types of errors that arise by hitting the wrong key from that intended when entering a drug dosage in a typical hospital device. Some errors simply replace one digit or decimal point with an incorrect digit or decimal point. Some errors though are a termination slip where a wrong key effectively terminates the number entry. The proportion of termination slips to incorrect digit errors is likely to be substantially greater than 1 as many devices take *any* key press other than a digit or a decimal point to terminate number entry and process the number.

### 4.1. Defining errors

We define *valid numbers* as those meeting the form described by the ISMP, as outlined in table 3, with the additional rules—which the ISMP does not mention—that multiple decimal points are invalid, and leading zeros, except for numbers less than 1, are invalid. One might also consider rules about valid ranges of numbers and for significant figures, though doing so is beyond the scope of the present paper.

**Table 3.** Selected entries from the Institute for Safe Medication Practices (2006) *List of error-prone abbreviations, symbols and dose designations*. Example errors that ‘… should NEVER be used when communicating medical information’ [their emphasis]. Reproduced with permission. Copyright © ISMP 2009.

dose designations	intended meaning	misinterpretation	correction
trailing zero after decimal point (e.g. 1 • 0 mg)	1 mg	mistaken as 10 mg if the decimal point is not seen	do not use trailing zeros for doses expressed in whole numbers
‘Naked’ decimal point (e.g. • 5 mg)	0 • 5 mg	mistaken as 5 mg if the decimal point is not seen	use zero before a decimal point when the dose is less than a whole unit
large doses without properly placed commas (e.g. 100000 units; 1000000 units)	100, 000 units 1, 000, 000 units	100000 has been mistaken as 10, 000 or 1, 000, 000; 1000000 has been mistaken as 100, 000	use commas for dosing units at or above 1, 000, or use words such as 100 ‘thousand’ or 1 ‘million’ to improve readability

Not all errors result in serious problems: an erroneously entered value of 1 • 3 is probably fine if the intended value is 1 • 31. Thus, we define the notion of *out by r errors* where the ratio of the original intended number to the final processed number acted on or vice versa is at least *r*. Specifically, if the intended number is *i* and the number finally acted on is *a*, then there is an out by *r* error if *i*/*a* ≥ *r* or *a*/*i* ≥ *r*. Obviously, out by *r* errors are only errors when *r >* 1.

For instance, if the intended number is 20, ending up with 2 or less or ending up with 200 or more are both out by 10 errors, and in fact both are out by *r* errors for any *r* less than 10 as well—they are both out by 2 errors, since the number processed or acted on in both cases is at least a factor of 2 out from the intended value of 20. We are careful to say ‘the number processed or acted on’ since the number entered by the user may be one thing, but the device may misinterpret it (for instance, when there is a keying error): what matters, whether there is an out by *r* error, is the final number acted on compared with the intended number, not what the user entered nor what they thought they entered.

Whether harm or other unwanted outcomes arise from an out by *r* error is another matter. For example, an out by 2 error in an intravenous chemotherapy drug would probably be far more serious than an out by 10 error in counting vitamin C pills, as well as being harder to notice at the time the numerical error occurred; in contrast, in accountancy, *any* out by *r* error warrants investigation. However, out by 10 errors are very likely to occur with decimal point or termination slips (e.g. keying too few or too many digits), and they are widely recognized as leading to adverse outcomes in healthcare (Lesar 2002).

### 4.2. Analysis methods

The purpose of the analysis is to see the extent to which syntax checking reduces out by 10 errors when entering numbers in a typical interactive device. The main problem in conducting this analysis is that there are no easily available data on the kinds of errors made when doing number entry, the underlying rate of error, or the distribution of numbers being entered. The analysis therefore takes an exhaustive approach looking at all possible number entries within a given range.

This is a somewhat unconventional approach to user interface evaluation, as it is done without human participants—the unforced human error rates are so low and the probability of an error coinciding with a noticeable design defect so low that conventional evaluation could not have produced reliable results within reasonable resources. We also note that well over 30 years of conventional user interface evaluation performing empirical experiments with users (Johnson 2006) has failed to spot, let alone evaluate, the problems this paper addresses.

We first analysed the impact of syntax checking using a Monte Carlo method. The Monte Carlo method is both conceptually simple and simple to program, and it is therefore likely to be valid because any errors in its programming should be reasonably obvious. However, because our initial results were surprising, we chose to program two other approaches independently, using quite different techniques, and using two different programmers working independently.

We thus programmed three independent analyses:

— a Monte Carlo simulation of number entry with varying error rates;
— an exhaustive method where each target number in the range is considered in turn;
— a symbolic analysis where the proportion of blocked out by *r* errors is calculated as a function of the underlying keystroke error rates.

The Monte Carlo method and the symbolic analysis were both written in Mathematica by H.T., and the exhaustive method was written independently in Java by P.C., making use of Microsoft Excel for convenient data presentation. (All program source code is available in the electronic supplementary material; the Mathematica code is an interactive ‘notebook’ and provides detailed explanations, and options to analyse different number-parsing strategies.) In all methods, the general approach was to consider a number intended to be keyed, simulate user slips in the keying of that number and then parse the resultant key sequence to assign it a value, as a typical device might do (we explored various ways of parsing numbers, covering the cases illustrated in table 1). We are thus able to estimate the probability of out by 10 errors and the probability of out by 10 errors that would be blocked by syntax checks.

#### 4.2.1. Parametrizing error probabilities

For a given single key-press when entering numbers, we use *e* to denote the probability that any key other than the intended key was pressed. From other contexts, it can be inferred that *e* is at most 0 • 1, but it is more likely to be between 0 • 01 and 0 • 05. However in the case of high stress such as users may encounter during surgery, *e* may be somewhat higher; then again, with trained and relaxed professionals, *e* may be somewhat lower (Wickens & Hollands 2000). The only certainty is that *e* is greater than zero as human error is inevitable.

Sometimes, the incorrect key pressed has the effect of terminating the number entry, for example, by mistakenly pressing an 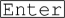 or 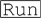 key, which would make the device accept the entered number and start functioning. In other cases, the incorrect key is simply the wrong digit or a decimal point and number entry is able to continue.

We denote the probability of miskeying but not terminating the number entry by *p* and the probability of incorrect termination errors by *q*. Clearly, *e* = *p* + *q*, but in order to simplify the variations in *p* and *q* while ensuring 0 ≤ *e* ≤ 1, we define *k* = *q*/*p*, that is, *k* is the proportion of termination to non-termination errors. Given that in many devices we considered there are many keys that when pressed cause a termination of number entry, we believe that 0 • 5 ≤ *k* ≤ 10 is a realistic range for *k*.

#### 4.2.2. The Monte Carlo method

The simplest approach is to use a Monte Carlo method, as it closely models how a user would enter a number: a certain number is intended but keying errors mean that another number may be keyed in. Thus, the Monte Carlo program randomly selects a number from 0 • 01 to 99 • 99 in increments of 0 • 01, works out the correct key sequence to enter this number and then uses the parameters *p* and *q*, as described above, to randomly alter the key sequence with the appropriate probabilities. The key sequence is then parsed according to the device being simulated and given a value. The sequences are marked as to whether they would produce an out by 10 error and whether syntax checking as we propose would have blocked it.

Because there are many numbers to sample and the probabilities of error are relatively low, the Monte Carlo method has to be run for a long time to obtain accurate results. Of course, the longer it is run, the more accurate the results will be.

**Figure 3.**
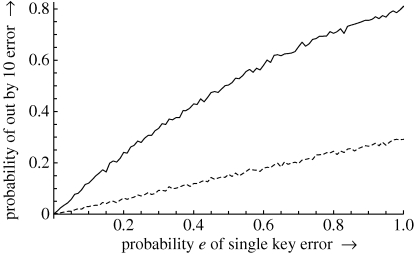
A plot of the probabilities of an out by 10 error and a blocked out by 10 error as found in a 5000 sample Monte Carlo method as a function of *e* for *k* = 10. The solid line shows the behaviour of a calculator-type device (all but the first decimal point is ignored); the dashed line shows the reduction in out by 10 errors by blocking syntax errors. Note that at certainty of error (*e* = 1), out by 10 errors are not certain; this is because *e* measures keying error rates, not out by 10 rates. (Some keying errors create numbers that are out by less than 10.)

A plot of results from a typical Monte Carlo analysis is shown in figure 3.

#### 4.2.3. Exhaustive simulation

In Monte Carlo, target numbers are chosen at random and sample the range; in contrast, in exhaustive simulation, *every* number from 0 • 01 to 99 • 99 in increments of 0 • 01 is treated in turn as the target number. The analysis then exhaustively considers every possible miskeying of the target number and calculates the probability of producing each erroneous entry. As before, the miskeyed entries are marked as to whether they would produce an out by *r* error (the program does not just consider out by 10 errors) and whether syntax checking would have blocked them. Thus, by summing the probabilities, it is possible to say what the probability of an out by 10 error is for a given target entry, and also what proportion of those errors would be blocked.

Data for selected values of *e* and *k* are given in table 5. For particular values of *k* it is notable that differences in *e* have only a small effect on the proportion of out by 10 errors that are blocked by syntax checking. Also, even for what we believe to be very low values of *k*, syntax checking still blocks around a third of all out by 10 errors. Both the program and full data are available in the electronic supplementary material.

#### 4.2.4. Symbolic method

Mathematica is a symbolic mathematics system that can be programmed, but it does not need to know values. For example, adding *p* + *q* + *p* in Mathematica will give the expression 2*p* + *q* as its result, whereas, in a conventional programming language, specific numerical values would be needed for both *p* and *q* before they could be added. The result in a conventional language would be numerically equal to 2*p* + *q* (within limits of precision), but would be a number, not an expression as it is in Mathematica. In particular, in Mathematica, values of *p* and *q* can even be assigned later, for instance to plot a graph, whereas in a conventional programming language, assigning different values to variables later would not change a previously calculated expression's value.

In Mathematica, we follow exactly the same procedure as in exhaustive simulation, except we do not need explicit values for *p* or *q*, the probabilities of keying error. The results are now formulas, for example 0 • 3*e* − 0 • 062*e*^2^ + 0 • 0061*e*^3^, here assuming *k* = 10 (fixing *k* is not necessary, but simplifies the formula considerably for illustrative purposes).

A disadvantage of Mathematica is that it is slower than Java, so we do not consider the same range of numbers as in the two previous methods but instead restrict the range to all values from 0 • 1 to 99 • 9 in increments of 0 • 1.

**Figure 4.**
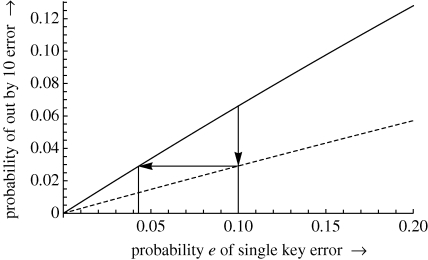
Plot of probability of an out by 10 error against probability of single key errors. As in figure 3, the solid line shows the behaviour of a simulated real device, and the dashed line shows the reduction in out by 10 errors by blocking syntax errors. The plot illustrates specifically how users with a given *e* (here 0 • 1) have their effective error rate approximately halved; since the graph is approximately linear for small *e*, this improvement in out by 10 rate would otherwise have to have been achieved by halving the user's keying error rate. The range of numbers covered in this plot is 0 • 1 to 99 • 9 with *k* = 10.

To draw graphs, each possible number parsed is considered and its out by *r* value calculated. The probability of each out by *r* value is accumulated in bins. At the end of a run, the bins contain the symbolic probabilities summed over a range (e.g. 9 • 995 ≤ *r <* 10 • 005) of out by *r* values. Figure 4 is an example of plotting such functions.

### 4.3. Summary of findings

Detailed insights specific to the particular analysis methods were discussed in the appropriate sections above. The results confirm that an error-blocking number entry system as we propose will reduce errors and out by *r* errors in particular, because some keying errors result in syntax errors that are given spurious values on typical devices. Normally, reducing error rates *e* by training or more careful checking becomes increasingly costly the smaller *e*, but error blocking has constant cost even as *e* goes to 0.

The probability of an out by 10 error of course depends heavily on the basic rate of miskeying. Nonetheless, our analysis (by all three methods) indicates that blocking invalid numbers results in roughly halving the probability of an out by 10 error and that the reduction is essentially independent of the underlying rate of miskeying. With reference to figure 4, we can see that the graph is approximately linear for small *e*: halving *e* approximately halves the rate of out by 10 errors.

## 5. Discussion

Our proposed user interface design approach is (i) to handle number entry consistently and (ii) to block errors immediately, and this is clearly appropriate for many applications. However, there are currently no data on the situated effectiveness of this or of alternative approaches.

Vicente *et al.* (2003) estimate the mortality rate for avoidable user programming errors for a patient-controlled analgesia (PCA) device to be 1 : 33 000 to 1 : 338 800 (by comparison, the mortality rate from anaesthesia is 1 : 200 000 to 1 : 300 000), with an absolute rate of 65–667 deaths per year in the USA for a *single* PCA model. A study by Lesar (2002) suggests that out by 10 errors in medication dosing occur in 1 per cent of hospital admissions and that 45 per cent of these result in serious or severe outcomes. Although Lesar's methodology involved pharmacy computers, he did not study user errors with drug delivery systems, so these figures underestimate number entry error in general.

Our approach, by approximately halving the probability of such errors, should have a valuable impact in saving lives. However, it is also possible that protecting users may encourage them to be more reckless; it is possible that blocking a single error would create anxiety or stress in the user that will increase their longer term error rates (for example, if the device beeps on detecting an error, it may make it embarrassingly obvious that the user is making an error, which may make the user more likely to make further errors). It may even be that automatically correcting erroneous input, even if not perfectly accurately, could be more effective for reducing overall error rates. Another possibility is that users would develop an error-prone work-around to avoid blocking; for example, rather than correcting 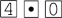, they might continue and press, say, 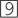, thus getting the valid number form 4 • 09. That would counteract the error warning, but almost certainly would not correct the underlying error.

Given the importance and ubiquity of dependable number entry, it is unfortunate that empirical evidence one way or the other is simply unavailable owing to inadequate device logs.

### 5.1. Supplementary approaches

The goal is to improve the reliability of number entry, but the focus of this paper on the advantages of error blocking does not exhaust useful approaches to improve reliability.

Often, displayed numbers have constant digit spacing regardless of any decimal point: for example, there is little visible difference between 35 and 3 • 5 when displayed on standard seven-segment displays as 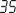 and 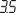; worse, 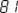 has an expanded spacing, even though there is no decimal point (see also table 4). Moreover, the standard, small decimal point aligned on the baseline is easy to obscure behind an escutcheon at poor viewing angles. Given the importance of decimal points, they should be made more salient. Safety-critical domains should avoid seven-segment and other limited resolution displays. If seven-segment displays must be used, it would be better to display a 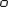 symbol for the decimal point, as in 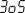. It would be far better to use higher resolution indicators.

**Table 4.** Calculators make decimal points inconspicuous and ignore over-long number entry. The table shows the results of calculating the 2009 US population as a proportion of world population on a typical handheld calculator, here a Canon LS-270H, compared with values correctly rounded for an eight-digit display. In *all cases*, the calculator displays an incorrect result, only in one case reporting an error, and then with a relatively inconspicuous marker. (Many calculators display a small ‘E’ instead of the full word.) Compare the standard inconspicuous seven-segment decimal point in the calculator results column against its clear presentation in the column of correct values.

calculation	keystrokes	result *drawn to scale*	correct value
percentage	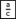 100 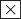 306900000 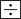 6707000000 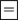	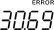	4 • 575 816 3
percentage	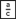 306900000 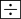 6707000000 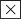 100 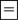	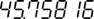	4 • 575 816 3
fraction	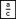 306900000 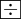 6707000000 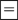	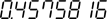	0 • 045 758 2

**Table 5.** Out by 10 probabilities for selected values of *e* and *k*. The table shows the probabilities of out by 10 errors blocked by syntactic checking, generated from an exhaustive analysis of errors when keying target numbers in the range 0 • 01–99 • 99 in steps of 0 • 01. The table shows clearly that even with very conservative assumptions, over 30% of out by 10 errors are blocked.

	*k*				
*e*	0 · 5	1 · 5	2 · 0	5 · 0	10 · 0
0 · 005	0 · 327	0 · 429	0 · 451	0 · 504	0 · 526
0 · 01	0 · 328	0 · 431	0 · 453	0 · 505	0 · 527
0 · 02	0 · 330	0 · 433	0 · 456	0 · 508	0 · 530
0 · 05	0 · 336	0 · 443	0 · 465	0 · 517	0 · 538
0 · 1	0 · 348	0 · 458	0 · 480	0 · 531	0 · 552

Decimal points should be larger, as used in our demonstration user interface and indeed as used throughout this paper. Decimal points can also be animated (e.g. displaying 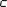, 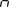, 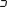, 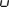 in turn), or numbers could be spoken back to the user in synthesized speech—which would also allow the voice to say ‘no decimal point’ as the case may be.

The international standard IEC 60062 defines a generalization of a decimal point, replacing it with a more-visible ‘radix point’. Thus, the conventional 4.7 k*Ω* (i.e. 4700 *Ω*) is written 4 k 7 *Ω*, replacing the decimal point with the more clearly visible multiplier, here the SI prefix k. The technique can be used with any units.

For some applications, it will be important to consider grouping (for instance, requiring a comma key as recommended by the ISMP, though the space is the appropriate SI symbol) as this will be likely to reduce undetected keying errors significantly, but at the cost of being unconventional. In some domains, appropriate choices of multipliers (k, m, etc.) can avoid commas to the left of the decimal point: requiring numbers to be entered in appropriate units would provide a further check that the number keyed is the number intended.

There are systems of ‘preferred numbers’. If numbers are known to be restricted to certain values (e.g. 1, 2, 5, 10, 20, 50, …), then the interactive system can check that numbers entered conform to those restrictions. There are established systems of preferred numbers, such as IEC 60063; for example, the E6 series (10, 15, 22, 33, 47, 68, …) has six preferred values covering any value to within 20 per cent. So-called dose error reduction systems typically restrict acceptable values to lie between given pre-defined limits, and again facilitate further checks, provided the ranges themselves are error-free.

Further methods can be found outside of technology; thus, improved training for operators or procedures such as buddy systems (i.e. where two or more operators must first agree) would help. Human factors should not be ignored: for example, tired users have higher error rates. Implementing human factors ideas will reduce the underlying rate of miskeying, but our method of blocking, being technology-oriented, is independent of and in addition to improvements to human factors—it would still halve the probability of any out by 10 errors even after human factors considerations had reduced *e*.

### 5.2. The tip of the iceberg

The problems considered here are also exacerbated in that users typically rely on other devices such as calculators to first work out what numbers need to be entered: numbers will be entered into a calculator or spreadsheet and then different numbers will be entered into another application, thus providing multiple opportunities for slips. Calculators are depended on by people who do not know correct arithmetic results, and therefore users are unlikely to detect incorrect or idiosyncratic results. Yet, it has long been known that calculators are badly designed and ill-defined (Thimbleby 2000). Specific to number entry, typical eight-digit calculators permit the apparent entry of longer numbers without alerting users to overflow, and hence cause out by 10 errors (table 4).

There are problems even in such simple number entry areas as entering times: some digital clocks permit any ‘time’ up to 99.99 to be entered, and some crash when numbers are entered that are not valid 12-hour times (Thimbleby & Witten 1993).

This paper has considered only those errors where users make keying errors within the set of numeric characters, or that are due to late or premature termination of a number. In general, however, user interfaces provide many other keys, and user errors may extend to incorrect pressing of *any* key. For example, a user may press the erroneous sequence 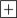 then 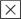 on a calculator, but typically it will be parsed as having some ‘valid’ meaning, idiosyncratic to the particular make, model and version of the calculator. The user may remain unaware of their slip, and therefore unaware that their calculation is incorrect. More obviously, if the user substitutes one operator for another in a slip (e.g. keying 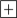 instead of 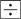), the calculator simply performs an unintended calculation. *Normal general purpose calculators are inadequate for any safety-critical application*.

There are also alternative number entry mechanisms that bring their own particular problems, again without consideration of the certainty that users occasionally make errors. Up and down arrows to increment or decrement a value can have ‘wrap-around’—for instance, if a number goes above a certain value, say 99, then it wraps back to 0. A single incorrect key press drastically alters the value of what was entered from what was intended. Even when numbers are entered using a single press to cycle between preset values, the design of a device can be such that it triggers unseen changes in the device. Exactly this method of number entry on a blood pressure *monitoring* machine nearly killed a patient in surgery (Degani 2001).

Number problems are not restricted just to user interfaces of interactive systems, but extend into the programming languages that implement the interfaces. Providing dependable number user interfaces *requires* a reliable programming language to implement the user interface in.

Consider, for instance, that in JavaScript parseFloat(″1.2.3″) gives the incorrect value 1 • 2 with no error reported; and parseInt(″08″), as might be used to handle input from a user trying to enter the month of August, gives the surprising value 0, again without error. Java, another popular modern programming language, has no reasonable way to input numbers, and the methods it does provide have *ad hoc* exceptions to what they can handle (Arnold & Gosling 1996): evidently, dependable number input was not a design goal.

Critically analysing programming languages would take us beyond the scope of this paper, but suffice to say that if the languages themselves are unreliable—as they are—dependable user interfaces will be hard to construct even for the most experienced programmers.

### 5.3. General purpose number entry and what it should do

It is regrettable that there is not a standard number entry algorithm available, with appropriate error handling, as at least part of the problem arises from programmers reinventing number parsing algorithms on a case-by-case basis and ignoring the complexities of handling exceptions in their chosen languages. Designing a standard algorithm that is robust in the face of the defects of many programming languages will be a challenging task.

Having detected problems, what should the interface do with them? Under different assumptions, there will be different trade-offs between ignoring certain user errors, to correct some, and to warn of others. In some domains, it will be reasonable to correct a user keying 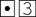 to ‘0 • 3’, whereas under the ISMP rules, it is not appropriate (what if the user meant 3 and the decimal point was a slip?). The key trade-off is to balance what must be forbidden and what can be corrected against the dependability and usability requirements of the domain. A full discussion of the trade-offs is beyond the scope of this paper, but clearly any general purpose solution to number entry will have to be configurable.

As an example of a different domain with different rules, consider the numbers entered into personal bank accounts, which never have more than two digits after the decimal point. Although preventing out by 10 errors is important, ‘out by *r* errors’ as such are less pertinent to error analysis. Some bank cash dispensing machines have a decimal point key that does nothing, yet they show monetary values with two decimal digits: users are expected to enter numbers always followed by two zeros. ISMP rules do not apply to such monetary numbers (for example, 3 • 50 is a valid monetary value, but is not a valid drug dose because of the trailing zero).

However, what is not acceptable, for almost all domains, assumptions and trade-offs, is current common practice: to ignore user errors and then act unpredictably.

## 6. Concluding Remarks

We have shown that user errors are ignored or worse by many number entry systems in user interfaces from interactive devices to desk-top applications; in all domains, this causes confusion and problems, possibly leading to harm. Although many strategies can improve error rates, such as better user training, our findings suggest that improving user interfaces to prevent badly formed numbers can halve the probability of out by 10 errors. The improvement is available to all users at essentially no further cost, since it does not affect already correct number entry.

Further work is suggested. For example, we have shown in this paper theoretical and engineering grounds for improving number entry procedures. In ecological settings, it is possible that error blocking will have unwanted consequences. For example, operators thinking that a device is safer may take more risks, and some of those risks may result in consequences the error blocking is unable to mitigate. Another possibility is that blocking errors will increase stress (a ‘beep’ might embarrassingly draw bystanders' attention to the user's errors), thus ironically increasing the baseline rate of errors. Although it appears that blocking errors as illustrated in figure 2 would be an essential improvement in many contexts, exactly how best errors may be blocked (audibly, visually, confidentially, etc.) in order to achieve the best outcomes in the domain of use is yet to be established with confidence. In practical terms, it seems essential to develop a formal specification of a better user interface for number entry (including reliable logging), and it seems essential to devise practical ways of ensuring that systems conform to that standard. The practical ways would involve formal methods, code inspection, as well as error testing, much as in this paper we tested the behaviour of devices with 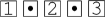. It would not be unreasonable to generalize the concepts to time entry, date entry and other areas (e.g. social security numbers, surnames, post codes, etc.) by using grammars, though to do so takes us beyond the scope of the present paper.

At a far more basic level, very little is documented in the literature about the types of errors people make and the frequency with which they make them in any form of number entry task. Even less is known about the interaction of error rates with varying user interface designs. Systematic gathering of number entry performance varying from experimental-style set-ups to naturalistic observations would be invaluable in helping to assess the causes and patterns of errors, and would therefore suggest new ways to reduce errors, or to estimate the harm caused and the economic or social value of blocking errors as we propose.

One of the most strategic places to start to improve number entry would be to improve the underlying facilities provided in programming languages. In the short run, libraries should be defined to provide definitive and reliable number entry; in the longer run, new languages should be defined to be dependable for user interaction—it is astonishing that the designers of programming languages are so keen on brevity and backwards compatibility that dependability is knowingly sacrificed.

Although many systems such as Excel are scriptable (i.e. they can be programmed by end users), it is impossible to circumvent underlying dependability defects simply by adding more code without having appropriate built-in features: unfortunately, then, many application programs will need major revision to be more dependable (discussing the possible merits in this regard for open source approaches is beyond the scope of this paper). Certainly, programmers need to be aware that dependable number entry is not a trivial problem, and even the programming languages they are using need careful checking.

While the flexibility and power of Excel can be considered a virtue, it is surprising that Excel and similar programs do not have a mode where dependability (in this case, WYSIWYG—‘what you see is what you get’) can be rigorously enforced. Many programs, we argue, could be improved by introducing an ‘honesty’ mode that restricts the features that can be used, whether accidentally or deliberately, to limit the scope for creating misleading results.

Users and their managers need to be aware that number entry is not as dependable as is commonly assumed: keying errors may remain undetected and may result in erroneous numbers being processed without warning. Independent checks should be used for safety-critical number entry (this is a standard practice in hospitals, where two nurses should independently check calculations), but, moreover, the independent checks should not be restricted to just the calculations themselves, but must extend to the values actually processed by the devices—to check whether the device itself is registering the intended value. Our research shows that commonly it may not be. Users and their managers and, where appropriate, their legal representatives also need to be aware that device logs may also be misleading.

Finally, we have provided a working demonstration user interface that may be used as a model of better practice for more dependable interactive number entry.
